# Synthesis and structure of norfloxacinium acetate sesquihydrate

**DOI:** 10.1107/S2056989026005670

**Published:** 2026-06-02

**Authors:** Abdusamat Rasulov, Batirbay Torambetov, Aziza Erejepova, Umidjon Annaev, Jabbor Suyunov, Bekmurod Alimnazarov, Jamshid Ashurov

**Affiliations:** a Termez University of Economics and Service, 41B Farovon St, Termiz, 190111, Uzbekistan; b National University of Uzbekistan named after Mirzo Ulugbek 4 University St Tashkent 100174 Uzbekistan; c Termez State University, Barkamol Avlod St 43, Termez, 190111, Uzbekistan; d Institute of Bioorganic Chemistry, Academy of Sciences of Uzbekistan, M. Ulugbek, St, 83, Tashkent, 100125, Uzbekistan; University of Aberdeen, United Kingdom

**Keywords:** crystal structure, norfloxacin, mol­ecular structure, hydrogen bonds

## Abstract

The title hydrated salt, C_16_H_19_FN_3_O_3_^+^·C_2_H_3_O_2_^−^·1.5H_2_O, crystallizes with two cations, two anions and three water mol­ecules of crystallization in the asymmetric unit. The protonation of the piperazine secondary amine group of norfloxacin occurs *via* proton transfer from acetic acid. In the extended structure, the components are linked into chains propagating along the *a*-axis direction through numerous N—H⋯O and O—H⋯O hydrogen bonds. Hirshfeld surface analysis and two-dimensional fingerprint plots confirm the significant contribution of H⋯O inter­actions to the consolidation of the crystal structure.

## Abstract 2

The components of the title mol­ecular salt are linked by numerous N—H⋯O and O—H⋯O hydrogen bonds and aromatic π–π stacking inter­actions.

## 1. Chemical context

Fluoro­quinolones (FQs) are among the most widely used classes of anti­microbial agents, with broad therapeutic applicability in the treatment of respiratory, urinary tract, gastrointestinal, and gynecological infections (Abidi *et al.*, 2016 ▸). Among them, norfloxacin, C_16_H_18_FN_3_O_3_, is a well-known fluoro­quinolone with broad-spectrum anti­biotic activity effective against both Gram-positive and Gram-negative bacteria (Grangé *et al.*, 1998 ▸). Structurally, norfloxacin contains a basic piperazinyl nitro­gen atom and a carb­oxy­lic acid functional group, which contribute to its versatile chemical behavior. In addition to its anti­bacterial activity, different solid forms of norfloxacin also demonstrate a range of biological properties, including anti­cancer, anti­viral, anti­oxidant, and anti­fungal activities (Barry *et al.*, 1984 ▸; Grangé *et al.*, 1998 ▸; Jiang *et al.*, 2025 ▸; Pandeya *et al.*, 2000 ▸; Goldstein, 1987 ▸; Ferrazzi *et al.*, 1988 ▸; Zeng *et al.*, 2024 ▸). Under appropriate conditions, protonation of the piperazinyl nitro­gen atom can occur, while the carb­oxy­lic acid group may undergo deprotonation, resulting in a zwitterionic form of the mol­ecule. This structural feature also enables norfloxacin to function as an effective ligand in metal complexation (Rasulov *et al.*, 2025 ▸). As part of our studies in this area, we report herein the synthesis and crystal structure of the title hydrated salt, C_16_H_19_FN_3_O_3_^+^·C_2_H_3_O_2_^−^·1.5H_2_O (**I**).
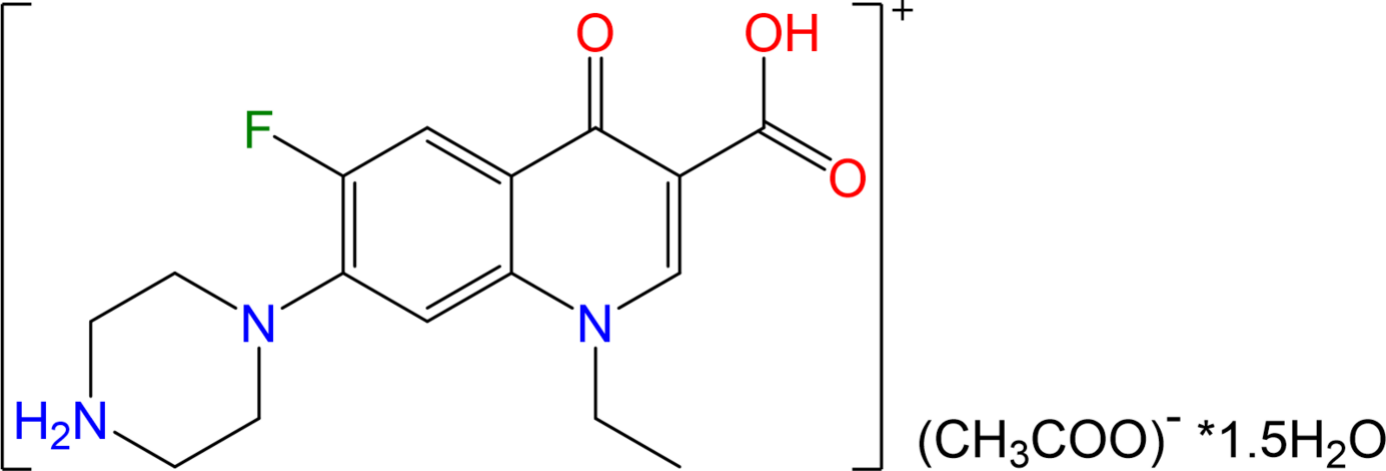


## 2. Structural commentary

**Figure 1:**
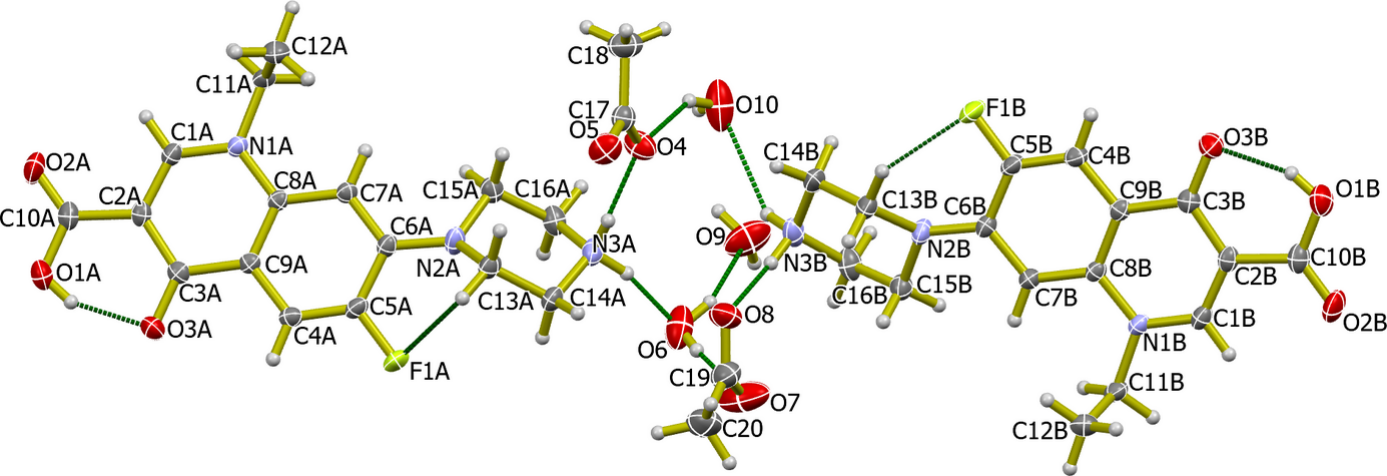
The mol­ecular structure of (**I**) with ellipsoids drawn at the 30% probability level. Weak interactions are shown as dotted lines.

Compound (**I**) crystallizes in the triclinic space group *P*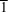. The crystal structure analysis reveals that the asymmetric unit comprises two norfloxacin cations (NF), two acetate anions and three water mol­ecules (Fig. 1 ▸). Equivalent atoms in the cations *A* and *B* are given suffixes A and B. In neutral norfloxacin, the piperazine N3 nitro­gen atom is typically protonated by a hydrogen atom originating from the carb­oxy­lic acid group, resulting in the formation of a zwitterionic species (*e.g.*, Gunnam & Nangia, 2023 ▸). However, in the crystal structure of compound (**I**), the hydrogen atom remains associated with the carb­oxy­lic acid moiety, while the N3 nitro­gen atom of the piperazine ring is protonated by a proton donated by an acetic acid mol­ecule. This assignment is supported by the significant differences observed in the C—O bond lengths: 0.115 Å between C10*A*—O1*A* and C10*A*—O2*A*, and 0.12 Å between C10*B*—O1*B* and C10*B*—O2*B*. In contrast, a delocalized carboxyl­ate group typically exhibits nearly equivalent C—O bond lengths, with differences of approximately 0.006 Å (Rasulov *et al.*, 2024 ▸). This characteristic delocalization is evident in the acetate anions present in the structure, where the C—O bond lengths are nearly identical: 1.244 (3) and 1.242 (3) Å for C17—O4 and C17—O5, respectively, and 1.212 (4) Å for C19—O7 and 1.221 (3) Å for C19—O8. The atoms of both the carboxyl and quinoline moieties are essentially coplanar: the maximum deviations from the mean plane are 0.035 (12) Å for atom C6A in cation *A* and 0.032 (12) Å for atom C3B in cation *B*. The dihedral angles between the carboxyl and quinoline planes are 2.00 (12)° for *A* and 1.46 (12)° for *B*, indicating near planarity, as expected. The nitro­gen atoms N2*A* and N2B, which are bonded to the quinoline rings, exhibit near-planar geometry, as indicated by the sums of the bond angles around them (353.3 and 353.1°, respectively). In contrast, the protonated nitro­gen atoms N3*A* and N3*B* display tetra­hedral geometries. The piperazine rings in both independent mol­ecules adopt chair conformations. Furthermore, the ethyl substituents attached to atoms N1*A* and N1*B* are oriented approximately perpendicular to the quinoline plane, as evidenced by the C1—N1—C11—C12 torsion angles of 94.8 (2) and 94.2 (2)°, respectively. Both cations feature an intra­molecular O—H⋯O hydrogen bond.

## 3. Supra­molecular features

**Figure 2:**
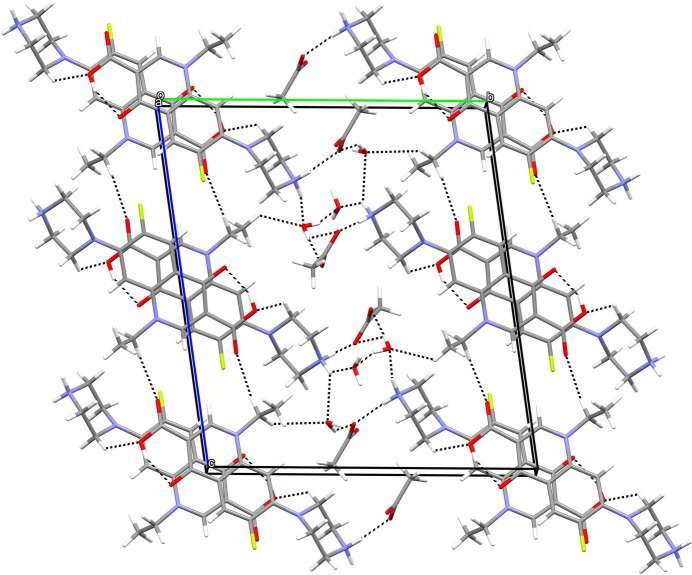
Visualization of the packing in (**I**) along the *a-*axis direction, showing hydrogen bonds as black dashed lines.

The packing of (**I**), as illustrated in Fig. 2 ▸, reveals that the acetate anions and water mol­ecules occupy inter­stitial sites between norfloxacinium cations. These species act as bridging units, linking the cations through numerous O—H⋯O and N—H⋯O hydrogen-bonding inter­actions (Table 1 ▸) into chains propagating along the *a*-axis direction. Weak C—H⋯O inter­actions further consolidate the structure. Aromatic π–π stacking inter­actions, with centroid–centroid separations ranging from 3.5395 (12) to 3.7393 (12) Å, arise from the overlap of aromatic rings of the norfloxacinium moieties.

## 4. Hirshfeld surface analysis

**Figure 3:**
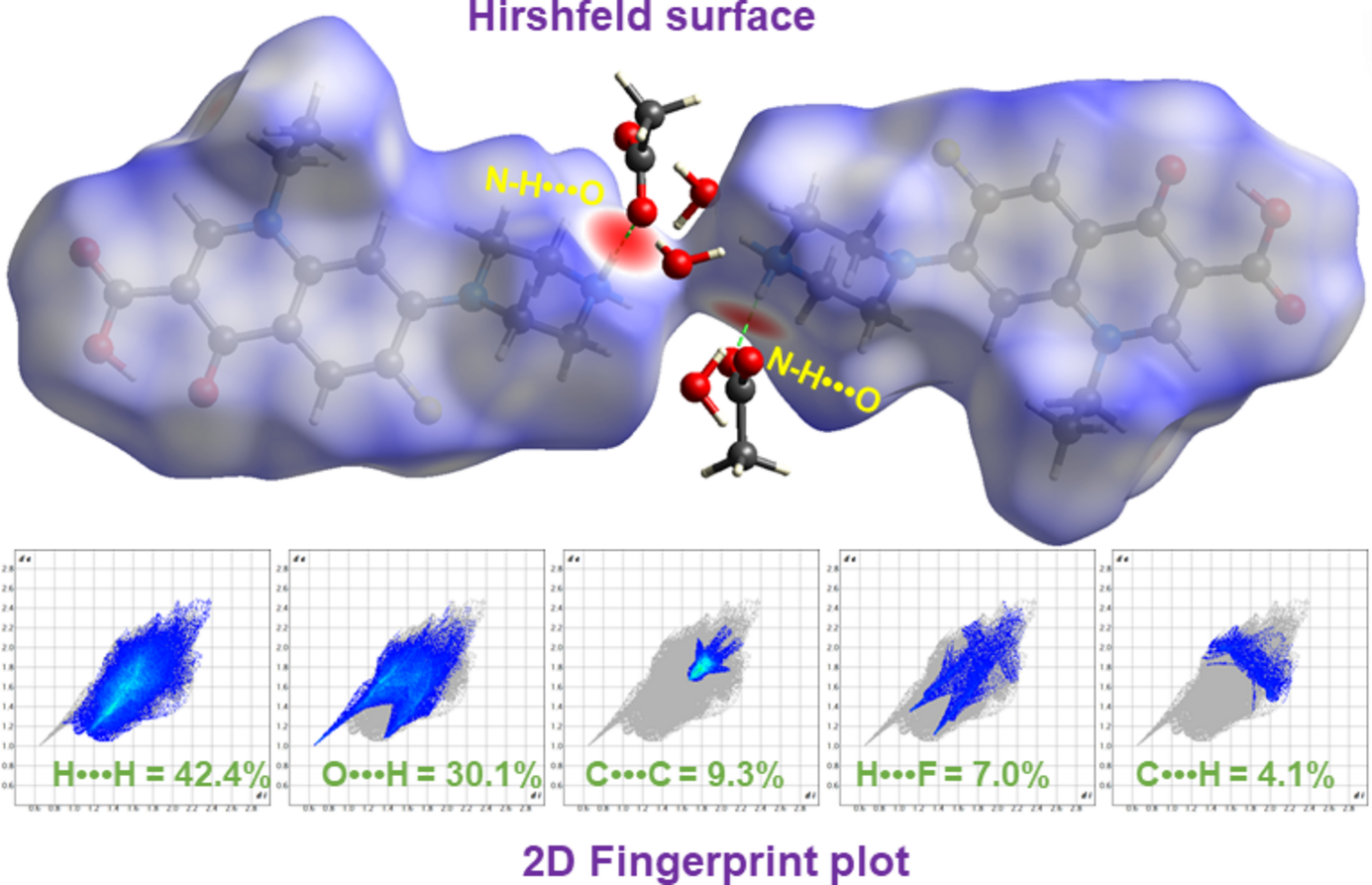
Hirshfeld surface and corresponding two-dimensional fingerprint plots for the norfloxacinium cations within (**I**) illustrating the contributions of different inter­molecular contacts to the overall Hirshfeld surface area.

Hirshfeld surface (HS) analysis and two-dimensional fingerprint plots were calculated using *CrystalExplorer* (Spackman *et al.*, 2021 ▸). The Hirshfeld surface (HS) of the norfloxacinium cation in (**I**) exhibits two prominent dark-red spots, indicating the presence of strong close contacts. These inter­actions are attributed to N–H⋯O hydrogen bonds, specifically between the protonated nitro­gen atom and the oxygen atoms of the acetate anion. The two-dimensional fingerprint plots qu­antify the contributions of various inter­molecular contacts to the Hirshfeld surface. The dominant inter­actions are H⋯H (42.4%), O⋯H/H⋯O (30.1%), C⋯C (9.3%), H⋯F/F⋯H (7.0%), C⋯H/H⋯C (4.1%), and C⋯O/O⋯C (3.1%), which together account for approximately 96.0% of the total surface area of the norfloxacinium cation in (**I**). The two-dimensional fingerprint plots further reveal that the O⋯H inter­actions are characterized by a distinct spike at *d*_i_ + *d*_e_ values of approximately 1.7 Å, indicative of strong hydrogen-bonding inter­actions (Fig. 3 ▸).

## 5. Database survey

A survey of the Cambridge Structural Database (CSD, Version 6.01, November 2025; Groom *et al.*, 2016 ▸) identified 85 crystal structures based on norfloxacin. Most of these structures incorporate water mol­ecules of crystallisation. Among these, only one structure contains norfloxacin together with both methanol and water (CSD refcode KEBROZ; Wang *et al.*, 2005 ▸). In contrast, six structures include norfloxacin along with additional components and two different types of solvent mol­ecules, namely water in combination with another solvent: three structures contain methanol (KEBGEH, KEBGAD, O’Malley *et al.*, 2022 ▸; KAHWAV, Jiao *et al.*, 2021 ▸), two contain aceto­nitrile (KEBQOB, O’Malley *et al.*, 2022 ▸; OFOZOC, Zhang *et al.*, 2025 ▸), and one contains ethanol (DONQIK; Zeng *et al.*, 2024 ▸). However, no crystal structure has been reported that contains a norfloxacinium cation, water mol­ecules, and acetate anions simultaneously within the same structure.

## 6. Synthesis and crystallization

31.9 mg (0.100 mmol) of NF was dissolved in 5 ml of a 0.1 *M* acetic acid solution. The resulting clear solution was stirred at room temperature for 30 minutes. The solution was then transferred to a vial with small holes in the lid to allow for evaporation. After about a week, block-like single crystals of the title salt were obtained.

## 7. Refinement

Crystal data, data collection and structure refinement details are summarized in Table 2 ▸. H atoms were positioned geometrically (N—H = 0.89, O—H =0.82– 0.85, C—H = 0.93–0.97 Å) and refined as riding with *U*_iso_(H) = 1.2–1.5*U*_eq_(C).

## Supplementary Material

Crystal structure: contains datablock(s) I. DOI: 10.1107/S2056989026005670/hb8223sup1.cif

Structure factors: contains datablock(s) I. DOI: 10.1107/S2056989026005670/hb8223Isup3.hkl

Supporting information file. DOI: 10.1107/S2056989026005670/hb8223Isup3.cml

CCDC reference: 2557861

Additional supporting information:  crystallographic information; 3D view; checkCIF report

## Figures and Tables

**Table 1:** Hydrogen-bond geometry (Å, °)

*D*—H⋯*A*	*D*—H	H⋯*A*	*D*⋯*A*	*D*—H⋯*A*
O1*A*—H1*A*⋯O3*A*	0.82	1.75	2.520 (2)	155
O1*B*—H1*B*⋯O3*B*	0.82	1.77	2.533 (2)	154
N3*A*—H3*AA*⋯O6	0.89	1.84	2.709 (3)	164
N3*A*—H3*AB*⋯O4	0.89	1.80	2.673 (3)	165
N3*B*—H3*BA*⋯O10	0.89	2.45	3.161 (4)	137
N3*B*—H3*BB*⋯O8	0.89	1.80	2.679 (3)	171
C13*B*—H13*C*⋯F1*B*	0.97	2.27	2.881 (3)	120
C13*A*—H13*A*⋯F1*A*	0.97	2.34	2.867 (3)	114
O6—H6*A*⋯O7	0.85	1.82	2.664 (5)	172
O6—H6*B*⋯O9	0.85	1.92	2.706 (5)	153
O9—H9*A*⋯O8^i^	0.85	1.91	2.706 (4)	156
O9—H9*B*⋯O5^i^	0.85	1.93	2.733 (3)	158
O10—H10*A*⋯O4	0.85	2.00	2.734 (3)	145
O10—H10*B*⋯O5^i^	0.85	1.99	2.811 (3)	161

Symmetry code: (i) 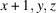.

**Table 2:** Experimental details

Crystal data	
Chemical formula	2C_16_H_19_FN_3_O_3_^+^·2C_2_H_3_O_2_^−^·3H_2_O
*M*_r_	812.82
Crystal system, space group	Triclinic, *P* 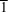
Temperature (K)	293
*a*, *b*, *c* (Å)	6.9746 (2), 15.8203 (5), 17.7430 (4)
α, β, γ (°)	81.803 (2), 88.257 (2), 85.917 (2)
*V* (Å^3^)	1932.44 (9)
*Z*	2
Radiation type	Cu *K*α
μ (mm^−1^)	0.96
Crystal size (mm)	0.2 × 0.18 × 0.14

Data collection	
Diffractometer	XtaLAB Synergy, Single source at home/near, HyPix3000
Absorption correction	Multi-scan (*CrysAlis PRO*; Rigaku OD, 2021 ▸)
*T*_min_, *T*_max_	0.744, 1.000
No. of measured, independent and observed [*I* > 2σ(*I*)] reflections	18863, 7459, 5848
*R*_int_	0.025
(sin θ/λ)_max_ (Å^−1^)	0.615

Refinement	
*R*[*F*^2^ > 2σ(*F*^2^)], *wR*(*F*^2^), *S*	0.057, 0.173, 1.06
No. of reflections	7459
No. of parameters	529
H-atom treatment	H-atom parameters constrained
Δρ_max_, Δρ_min_ (e Å^−3^)	0.52, −0.41

Computer programs: *CrysAlis PRO* (Rigaku OD, 2021 ▸), *SHELXT2014/5* (Sheldrick, 2015*a* ▸), *SHELXL2016/6* (Sheldrick, 2015*b* ▸) and *OLEX2* (Dolomanov *et al.*, 2009 ▸).

## supplementary crystallographic information

### 4-(3-Carboxy-1-ethyl-6-fluoro-4-oxo-1,4-dihydroquinolin-7-yl)piperazin-1-ium acetate sesquihydrate Crystal data

**Table:**

2C_16_H_19_FN_3_O_3_^+^·2C_2_H_3_O_2_^−^·3H_2_O	*Z* = 2
*M**_r_* = 812.82	*F*(000) = 860
Triclinic, *P*1	*D*_x_ = 1.397 Mg m^−^^3^
*a* = 6.9746 (2) Å	Cu *K*α radiation, λ = 1.54184 Å
*b* = 15.8203 (5) Å	Cell parameters from 8364 reflections
*c* = 17.7430 (4) Å	θ = 2.5–71.0°
α = 81.803 (2)°	µ = 0.96 mm^−^^1^
β = 88.257 (2)°	*T* = 293 K
γ = 85.917 (2)°	Block, colourless
*V* = 1932.44 (9) Å^3^	0.2 × 0.18 × 0.14 mm

### 4-(3-Carboxy-1-ethyl-6-fluoro-4-oxo-1,4-dihydroquinolin-7-yl)piperazin-1-ium acetate sesquihydrate Data collection

**Table:**

XtaLAB Synergy, Single source at home/near, HyPix3000 diffractometer	7459 independent reflections
Radiation source: micro-focus sealed X-ray tube, PhotonJet (Cu) X-ray Source	5848 reflections with *I* > 2σ(*I*)
Mirror monochromator	*R*_int_ = 0.025
Detector resolution: 10.0000 pixels mm^-1^	θ_max_ = 71.5°, θ_min_ = 2.5°
ω scans	*h* = −7→8
Absorption correction: multi-scan (CrysAlisPro; Rigaku OD, 2021)	*k* = −19→18
*T*_min_ = 0.744, *T*_max_ = 1.000	*l* = −21→21
18863 measured reflections	

### 4-(3-Carboxy-1-ethyl-6-fluoro-4-oxo-1,4-dihydroquinolin-7-yl)piperazin-1-ium acetate sesquihydrate Refinement

**Table:**

Refinement on *F*^2^	Primary atom site location: dual
Least-squares matrix: full	Hydrogen site location: mixed
*R*[*F*^2^ > 2σ(*F*^2^)] = 0.057	H-atom parameters constrained
*wR*(*F*^2^) = 0.173	*w* = 1/[σ^2^(*F*_o_^2^) + (0.0889*P*)^2^ + 0.5189*P*] where *P* = (*F*_o_^2^ + 2*F*_c_^2^)/3
*S* = 1.06	(Δ/σ)_max_ = 0.001
7459 reflections	Δρ_max_ = 0.52 e Å^−^^3^
529 parameters	Δρ_min_ = −0.41 e Å^−^^3^
0 restraints	

### 4-(3-Carboxy-1-ethyl-6-fluoro-4-oxo-1,4-dihydroquinolin-7-yl)piperazin-1-ium acetate sesquihydrate Special details

**Table:**

Geometry. All esds (except the esd in the dihedral angle between two l.s. planes) are estimated using the full covariance matrix. The cell esds are taken into account individually in the estimation of esds in distances, angles and torsion angles; correlations between esds in cell parameters are only used when they are defined by crystal symmetry. An approximate (isotropic) treatment of cell esds is used for estimating esds involving l.s. planes.

### 4-(3-Carboxy-1-ethyl-6-fluoro-4-oxo-1,4-dihydroquinolin-7-yl)piperazin-1-ium acetate sesquihydrate Fractional atomic coordinates and isotropic or equivalent isotropic displacement parameters (Å^2^)

**Table:**

	*x*	*y*	*z*	*U*_iso_*/*U*_eq_	
F1A	0.2271 (2)	0.10803 (9)	0.21519 (6)	0.0656 (4)	
F1B	0.2578 (2)	0.90602 (9)	0.28426 (7)	0.0701 (4)	
O3A	0.1952 (2)	−0.11888 (9)	0.04641 (9)	0.0546 (4)	
O3B	0.2017 (2)	1.13549 (9)	0.45354 (9)	0.0569 (4)	
N1A	0.3160 (2)	0.10123 (10)	−0.09290 (8)	0.0393 (3)	
O1A	0.1938 (3)	−0.19340 (10)	−0.06997 (10)	0.0630 (4)	
H1A	0.184965	−0.183275	−0.025899	0.095*	
N1B	0.3139 (2)	0.90822 (10)	0.59231 (8)	0.0410 (4)	
O1B	0.1845 (3)	1.20998 (11)	0.57105 (11)	0.0679 (5)	
H1B	0.177109	1.200554	0.527005	0.102*	
N2A	0.2993 (3)	0.25701 (12)	0.12489 (10)	0.0539 (5)	
N2B	0.3319 (3)	0.75145 (11)	0.37614 (10)	0.0514 (4)	
O2A	0.2583 (3)	−0.12359 (12)	−0.18336 (10)	0.0757 (5)	
O4	0.2323 (3)	0.53324 (12)	0.13304 (12)	0.0752 (5)	
O2B	0.2360 (3)	1.13513 (12)	0.68401 (10)	0.0780 (5)	
O5	−0.0768 (3)	0.52372 (14)	0.12313 (11)	0.0782 (5)	
N3A	0.3704 (3)	0.38585 (12)	0.21162 (11)	0.0560 (5)	
H3AA	0.431488	0.399232	0.251153	0.067*	
H3AB	0.305395	0.433043	0.189864	0.067*	
C9A	0.2517 (2)	0.02858 (12)	0.03328 (10)	0.0369 (4)	
C8A	0.2945 (2)	0.10365 (12)	−0.01480 (10)	0.0357 (4)	
C8B	0.2952 (2)	0.90688 (12)	0.51440 (10)	0.0370 (4)	
C9B	0.2545 (2)	0.98432 (12)	0.46637 (10)	0.0379 (4)	
O8	0.1701 (3)	0.49489 (13)	0.35463 (12)	0.0856 (6)	
N3B	0.4308 (3)	0.60690 (12)	0.30312 (11)	0.0627 (5)	
H3BA	0.508044	0.585644	0.268562	0.075*	
H3BB	0.354762	0.566199	0.322928	0.075*	
C6A	0.2827 (3)	0.18372 (13)	0.09223 (11)	0.0410 (4)	
C7A	0.3106 (3)	0.18033 (12)	0.01525 (10)	0.0386 (4)	
H7A	0.340474	0.229429	−0.017112	0.046*	
C7B	0.3154 (3)	0.82937 (12)	0.48467 (10)	0.0403 (4)	
H7B	0.340104	0.778641	0.517418	0.048*	
C3A	0.2318 (3)	−0.05117 (12)	0.00358 (11)	0.0408 (4)	
C2A	0.2563 (3)	−0.04716 (13)	−0.07723 (11)	0.0422 (4)	
C4A	0.2272 (3)	0.03187 (13)	0.11181 (10)	0.0423 (4)	
H4A	0.200490	−0.017159	0.144881	0.051*	
C6B	0.2994 (3)	0.82664 (13)	0.40730 (11)	0.0418 (4)	
C2B	0.2491 (3)	1.05993 (13)	0.57697 (11)	0.0436 (4)	
C3B	0.2324 (3)	1.06567 (12)	0.49635 (11)	0.0422 (4)	
C1B	0.2878 (3)	0.98236 (13)	0.62046 (11)	0.0445 (4)	
H1BA	0.296616	0.981076	0.672809	0.053*	
C1A	0.2947 (3)	0.02830 (13)	−0.12103 (11)	0.0428 (4)	
H1AA	0.306757	0.029063	−0.173459	0.051*	
C5A	0.2427 (3)	0.10651 (14)	0.13895 (10)	0.0445 (4)	
C4B	0.2389 (3)	0.98184 (13)	0.38799 (11)	0.0443 (4)	
H4B	0.212894	1.032166	0.354859	0.053*	
C5B	0.2619 (3)	0.90562 (14)	0.36073 (10)	0.0459 (4)	
C13A	0.1453 (3)	0.28851 (14)	0.17340 (11)	0.0493 (5)	
H13A	0.062871	0.242774	0.191753	0.059*	
H13B	0.067847	0.334530	0.144400	0.059*	
C11A	0.3431 (3)	0.17965 (13)	−0.14694 (11)	0.0493 (5)	
H11A	0.399889	0.164110	−0.194072	0.059*	
H11B	0.431490	0.214231	−0.125760	0.059*	
C11B	0.3469 (3)	0.82843 (14)	0.64605 (11)	0.0487 (5)	
H11C	0.404138	0.841281	0.691903	0.058*	
H11D	0.436182	0.789170	0.623093	0.058*	
C15A	0.4185 (3)	0.32425 (14)	0.09158 (13)	0.0520 (5)	
H15A	0.340331	0.370435	0.063113	0.062*	
H15B	0.513823	0.302264	0.057078	0.062*	
O10	0.5646 (3)	0.61555 (18)	0.1305 (2)	0.1193 (10)	
H10A	0.469795	0.596598	0.110789	0.179*	
H10B	0.659860	0.581644	0.121437	0.179*	
O6	0.5910 (4)	0.39611 (18)	0.33261 (18)	0.1090 (9)	
H6A	0.506869	0.404931	0.366787	0.163*	
H6B	0.645614	0.442910	0.322921	0.163*	
C17	0.0784 (3)	0.55213 (14)	0.09908 (13)	0.0531 (5)	
C10B	0.2235 (3)	1.13691 (15)	0.61620 (14)	0.0557 (5)	
C10A	0.2379 (3)	−0.12345 (14)	−0.11555 (13)	0.0525 (5)	
C15B	0.4305 (3)	0.67673 (14)	0.41897 (13)	0.0538 (5)	
H15C	0.337474	0.639771	0.445470	0.065*	
H15D	0.512109	0.694182	0.456469	0.065*	
C13B	0.1983 (3)	0.72648 (15)	0.32242 (12)	0.0553 (5)	
H13C	0.127172	0.776901	0.296989	0.066*	
H13D	0.107168	0.689036	0.349571	0.066*	
C16A	0.5156 (3)	0.35635 (15)	0.15547 (15)	0.0579 (6)	
H16A	0.601180	0.310907	0.181027	0.070*	
H16B	0.592237	0.403440	0.134896	0.070*	
C14A	0.2320 (4)	0.32049 (15)	0.23964 (12)	0.0553 (5)	
H14A	0.131053	0.345461	0.270064	0.066*	
H14B	0.297794	0.273057	0.271602	0.066*	
O9	0.8405 (4)	0.5136 (3)	0.27560 (15)	0.1274 (11)	
H9A	0.936726	0.492552	0.301912	0.191*	
H9B	0.867824	0.501967	0.231058	0.191*	
C12B	0.1613 (4)	0.78664 (16)	0.66655 (13)	0.0618 (6)	
H12D	0.105828	0.772923	0.621306	0.093*	
H12E	0.073458	0.825207	0.689845	0.093*	
H12F	0.186273	0.735161	0.701559	0.093*	
C14B	0.3101 (4)	0.68100 (15)	0.26473 (13)	0.0642 (6)	
H14C	0.221888	0.661063	0.230960	0.077*	
H14D	0.391638	0.720451	0.234254	0.077*	
C16B	0.5497 (4)	0.62967 (16)	0.36460 (16)	0.0666 (7)	
H16C	0.650172	0.665141	0.342073	0.080*	
H16D	0.610662	0.577863	0.392199	0.080*	
C12A	0.1558 (4)	0.23150 (16)	−0.16366 (14)	0.0653 (6)	
H12A	0.178247	0.281177	−0.199823	0.098*	
H12B	0.101955	0.249023	−0.117443	0.098*	
H12C	0.067786	0.197322	−0.184365	0.098*	
O7	0.3490 (4)	0.4337 (3)	0.44396 (15)	0.1414 (12)	
C19	0.1935 (5)	0.4465 (2)	0.41421 (16)	0.0816 (8)	
C18	0.0819 (5)	0.6111 (2)	0.02576 (17)	0.0877 (9)	
H18A	0.164861	0.656058	0.029906	0.132*	
H18B	−0.045778	0.635463	0.014468	0.132*	
H18C	0.128918	0.579859	−0.014351	0.132*	
C20	0.0277 (6)	0.4020 (3)	0.4541 (2)	0.1067 (12)	
H20A	0.023375	0.346453	0.438543	0.160*	
H20B	−0.090181	0.435196	0.441045	0.160*	
H20C	0.044072	0.395760	0.508178	0.160*	

### 4-(3-Carboxy-1-ethyl-6-fluoro-4-oxo-1,4-dihydroquinolin-7-yl)piperazin-1-ium acetate sesquihydrate Atomic displacement parameters (Å^2^)

**Table:**

	*U*^11^	*U*^22^	*U*^33^	*U*^12^	*U*^13^	*U*^23^
F1A	0.0998 (10)	0.0691 (8)	0.0290 (6)	−0.0120 (7)	0.0079 (6)	−0.0097 (6)
F1B	0.1138 (12)	0.0670 (8)	0.0295 (6)	0.0038 (8)	−0.0074 (6)	−0.0108 (6)
O3A	0.0650 (9)	0.0426 (8)	0.0560 (9)	−0.0132 (6)	0.0041 (7)	−0.0025 (6)
O3B	0.0671 (9)	0.0444 (8)	0.0580 (9)	0.0063 (7)	−0.0061 (7)	−0.0071 (7)
N1A	0.0440 (8)	0.0437 (8)	0.0315 (7)	−0.0081 (6)	0.0039 (6)	−0.0082 (6)
O1A	0.0687 (10)	0.0456 (8)	0.0792 (11)	−0.0114 (7)	0.0000 (9)	−0.0205 (8)
N1B	0.0462 (8)	0.0467 (9)	0.0312 (7)	−0.0031 (7)	0.0011 (6)	−0.0093 (6)
O1B	0.0757 (11)	0.0530 (9)	0.0783 (12)	0.0058 (8)	−0.0010 (9)	−0.0256 (9)
N2A	0.0597 (10)	0.0572 (11)	0.0512 (10)	−0.0184 (8)	0.0184 (8)	−0.0262 (8)
N2B	0.0644 (11)	0.0491 (10)	0.0436 (9)	0.0052 (8)	−0.0096 (8)	−0.0190 (8)
O2A	0.1024 (14)	0.0727 (12)	0.0613 (11)	−0.0161 (10)	0.0016 (9)	−0.0366 (9)
O4	0.0624 (10)	0.0633 (11)	0.0952 (14)	−0.0036 (8)	−0.0108 (9)	0.0066 (9)
O2B	0.1050 (14)	0.0768 (12)	0.0595 (11)	0.0030 (10)	−0.0004 (9)	−0.0392 (9)
O5	0.0591 (10)	0.1017 (15)	0.0737 (12)	−0.0077 (9)	0.0117 (9)	−0.0139 (11)
N3A	0.0667 (11)	0.0508 (10)	0.0544 (10)	−0.0001 (8)	−0.0108 (9)	−0.0206 (8)
C9A	0.0323 (8)	0.0424 (9)	0.0362 (9)	−0.0039 (7)	0.0014 (7)	−0.0057 (7)
C8A	0.0325 (8)	0.0433 (10)	0.0319 (8)	−0.0047 (7)	0.0018 (6)	−0.0069 (7)
C8B	0.0354 (9)	0.0451 (10)	0.0314 (8)	−0.0045 (7)	0.0026 (7)	−0.0084 (7)
C9B	0.0343 (9)	0.0435 (10)	0.0368 (9)	−0.0023 (7)	0.0016 (7)	−0.0091 (7)
O8	0.1035 (15)	0.0771 (13)	0.0731 (12)	−0.0296 (11)	0.0000 (11)	0.0114 (10)
N3B	0.0771 (13)	0.0517 (11)	0.0641 (12)	−0.0118 (9)	0.0242 (10)	−0.0259 (9)
C6A	0.0401 (9)	0.0475 (10)	0.0377 (9)	−0.0066 (8)	0.0038 (7)	−0.0127 (8)
C7A	0.0409 (9)	0.0417 (9)	0.0340 (9)	−0.0063 (7)	0.0037 (7)	−0.0068 (7)
C7B	0.0451 (10)	0.0420 (10)	0.0340 (9)	−0.0032 (8)	−0.0005 (7)	−0.0057 (7)
C3A	0.0343 (9)	0.0427 (10)	0.0455 (10)	−0.0045 (7)	0.0003 (7)	−0.0054 (8)
C2A	0.0373 (9)	0.0453 (10)	0.0469 (10)	−0.0049 (8)	−0.0009 (8)	−0.0154 (8)
C4A	0.0443 (10)	0.0466 (10)	0.0349 (9)	−0.0065 (8)	0.0024 (7)	−0.0005 (8)
C6B	0.0427 (10)	0.0477 (10)	0.0366 (9)	−0.0007 (8)	−0.0007 (7)	−0.0127 (8)
C2B	0.0373 (9)	0.0483 (11)	0.0481 (11)	−0.0023 (8)	0.0044 (8)	−0.0182 (9)
C3B	0.0327 (9)	0.0466 (11)	0.0476 (10)	−0.0002 (7)	0.0014 (7)	−0.0096 (8)
C1B	0.0448 (10)	0.0550 (12)	0.0367 (9)	−0.0055 (8)	0.0025 (8)	−0.0167 (8)
C1A	0.0434 (10)	0.0515 (11)	0.0361 (9)	−0.0060 (8)	0.0010 (7)	−0.0139 (8)
C5A	0.0504 (11)	0.0553 (11)	0.0284 (9)	−0.0054 (9)	0.0040 (7)	−0.0083 (8)
C4B	0.0477 (10)	0.0478 (11)	0.0361 (9)	0.0007 (8)	−0.0029 (8)	−0.0030 (8)
C5B	0.0560 (11)	0.0540 (11)	0.0277 (9)	−0.0010 (9)	−0.0024 (8)	−0.0073 (8)
C13A	0.0529 (11)	0.0567 (12)	0.0405 (10)	−0.0041 (9)	0.0071 (8)	−0.0158 (9)
C11A	0.0653 (13)	0.0513 (11)	0.0324 (9)	−0.0150 (9)	0.0089 (8)	−0.0061 (8)
C11B	0.0612 (12)	0.0534 (12)	0.0309 (9)	0.0015 (9)	−0.0051 (8)	−0.0054 (8)
C15A	0.0521 (11)	0.0518 (12)	0.0544 (12)	−0.0099 (9)	0.0106 (9)	−0.0148 (9)
O10	0.0627 (12)	0.1161 (19)	0.199 (3)	−0.0055 (12)	0.0016 (16)	−0.091 (2)
O6	0.0963 (17)	0.1104 (19)	0.133 (2)	0.0145 (13)	−0.0382 (15)	−0.0637 (17)
C17	0.0538 (12)	0.0519 (12)	0.0546 (12)	0.0019 (9)	0.0034 (10)	−0.0147 (10)
C10B	0.0503 (12)	0.0565 (13)	0.0648 (14)	−0.0012 (10)	0.0039 (10)	−0.0266 (11)
C10A	0.0491 (11)	0.0518 (12)	0.0613 (13)	−0.0061 (9)	−0.0030 (9)	−0.0219 (10)
C15B	0.0617 (13)	0.0485 (12)	0.0530 (12)	0.0011 (10)	−0.0059 (10)	−0.0147 (9)
C13B	0.0691 (14)	0.0575 (13)	0.0429 (11)	−0.0042 (10)	−0.0072 (10)	−0.0181 (10)
C16A	0.0519 (12)	0.0501 (12)	0.0750 (15)	−0.0058 (9)	−0.0012 (10)	−0.0187 (11)
C14A	0.0738 (14)	0.0542 (12)	0.0400 (10)	−0.0019 (10)	0.0011 (10)	−0.0154 (9)
O9	0.0998 (18)	0.199 (3)	0.0768 (15)	0.0219 (19)	0.0200 (13)	−0.0157 (19)
C12B	0.0737 (15)	0.0604 (14)	0.0482 (12)	−0.0097 (11)	0.0042 (11)	0.0046 (10)
C14B	0.1024 (19)	0.0537 (13)	0.0397 (11)	−0.0181 (12)	0.0125 (11)	−0.0146 (10)
C16B	0.0580 (13)	0.0547 (13)	0.0916 (18)	−0.0018 (10)	0.0052 (12)	−0.0280 (13)
C12A	0.0877 (17)	0.0576 (14)	0.0484 (12)	−0.0036 (12)	−0.0082 (12)	0.0009 (10)
O7	0.110 (2)	0.218 (4)	0.0845 (17)	−0.037 (2)	−0.0192 (15)	0.035 (2)
C19	0.099 (2)	0.091 (2)	0.0563 (15)	−0.0226 (17)	−0.0116 (15)	−0.0045 (15)
C18	0.103 (2)	0.087 (2)	0.0665 (17)	0.0013 (17)	0.0024 (16)	0.0070 (15)
C20	0.130 (3)	0.111 (3)	0.079 (2)	−0.051 (2)	0.005 (2)	0.0091 (19)

### 4-(3-Carboxy-1-ethyl-6-fluoro-4-oxo-1,4-dihydroquinolin-7-yl)piperazin-1-ium acetate sesquihydrate Geometric parameters (Å, º)

**Table:**

F1A—C5A	1.357 (2)	C2B—C1B	1.367 (3)
F1B—C5B	1.357 (2)	C2B—C10B	1.484 (3)
O3A—C3A	1.259 (2)	C1B—H1BA	0.9300
O3B—C3B	1.257 (2)	C1A—H1AA	0.9300
N1A—C8A	1.395 (2)	C4B—H4B	0.9300
N1A—C1A	1.340 (2)	C4B—C5B	1.359 (3)
N1A—C11A	1.476 (2)	C13A—H13A	0.9700
O1A—H1A	0.8200	C13A—H13B	0.9700
O1A—C10A	1.323 (3)	C13A—C14A	1.503 (3)
N1B—C8B	1.396 (2)	C11A—H11A	0.9700
N1B—C1B	1.338 (2)	C11A—H11B	0.9700
N1B—C11B	1.478 (3)	C11A—C12A	1.507 (3)
O1B—H1B	0.8200	C11B—H11C	0.9700
O1B—C10B	1.325 (3)	C11B—H11D	0.9700
N2A—C6A	1.381 (2)	C11B—C12B	1.507 (3)
N2A—C13A	1.460 (3)	C15A—H15A	0.9700
N2A—C15A	1.445 (3)	C15A—H15B	0.9700
N2B—C6B	1.383 (2)	C15A—C16A	1.504 (3)
N2B—C15B	1.452 (3)	O10—H10A	0.8500
N2B—C13B	1.465 (3)	O10—H10B	0.8500
O2A—C10A	1.207 (3)	O6—H6A	0.8500
O4—C17	1.244 (3)	O6—H6B	0.8500
O2B—C10B	1.205 (3)	C17—C18	1.490 (4)
O5—C17	1.242 (3)	C15B—H15C	0.9700
N3A—H3AA	0.8900	C15B—H15D	0.9700
N3A—H3AB	0.8900	C15B—C16B	1.498 (3)
N3A—C16A	1.497 (3)	C13B—H13C	0.9700
N3A—C14A	1.488 (3)	C13B—H13D	0.9700
C9A—C8A	1.405 (3)	C13B—C14B	1.502 (3)
C9A—C3A	1.452 (3)	C16A—H16A	0.9700
C9A—C4A	1.407 (2)	C16A—H16B	0.9700
C8A—C7A	1.406 (3)	C14A—H14A	0.9700
C8B—C9B	1.405 (3)	C14A—H14B	0.9700
C8B—C7B	1.399 (3)	O9—H9A	0.8500
C9B—C3B	1.458 (3)	O9—H9B	0.8500
C9B—C4B	1.405 (3)	C12B—H12D	0.9600
O8—C19	1.221 (3)	C12B—H12E	0.9600
N3B—H3BA	0.8900	C12B—H12F	0.9600
N3B—H3BB	0.8900	C14B—H14C	0.9700
N3B—C14B	1.488 (3)	C14B—H14D	0.9700
N3B—C16B	1.489 (3)	C16B—H16C	0.9700
C6A—C7A	1.382 (2)	C16B—H16D	0.9700
C6A—C5A	1.415 (3)	C12A—H12A	0.9600
C7A—H7A	0.9300	C12A—H12B	0.9600
C7B—H7B	0.9300	C12A—H12C	0.9600
C7B—C6B	1.388 (3)	O7—C19	1.212 (4)
C3A—C2A	1.432 (3)	C19—C20	1.503 (5)
C2A—C1A	1.367 (3)	C18—H18A	0.9600
C2A—C10A	1.482 (3)	C18—H18B	0.9600
C4A—H4A	0.9300	C18—H18C	0.9600
C4A—C5A	1.350 (3)	C20—H20A	0.9600
C6B—C5B	1.409 (3)	C20—H20B	0.9600
C2B—C3B	1.428 (3)	C20—H20C	0.9600
			
C8A—N1A—C11A	121.32 (15)	C12A—C11A—H11A	109.3
C1A—N1A—C8A	120.06 (16)	C12A—C11A—H11B	109.3
C1A—N1A—C11A	118.35 (15)	N1B—C11B—H11C	109.4
C10A—O1A—H1A	109.5	N1B—C11B—H11D	109.4
C8B—N1B—C11B	121.49 (15)	N1B—C11B—C12B	111.00 (17)
C1B—N1B—C8B	119.84 (16)	H11C—C11B—H11D	108.0
C1B—N1B—C11B	118.49 (15)	C12B—C11B—H11C	109.4
C10B—O1B—H1B	109.5	C12B—C11B—H11D	109.4
C6A—N2A—C13A	120.89 (17)	N2A—C15A—H15A	110.2
C6A—N2A—C15A	122.30 (16)	N2A—C15A—H15B	110.2
C15A—N2A—C13A	112.09 (17)	N2A—C15A—C16A	107.69 (19)
C6B—N2B—C15B	120.62 (16)	H15A—C15A—H15B	108.5
C6B—N2B—C13B	121.53 (17)	C16A—C15A—H15A	110.2
C15B—N2B—C13B	111.00 (17)	C16A—C15A—H15B	110.2
H3AA—N3A—H3AB	107.8	H10A—O10—H10B	104.5
C16A—N3A—H3AA	109.0	H6A—O6—H6B	104.5
C16A—N3A—H3AB	109.0	O4—C17—C18	118.1 (2)
C14A—N3A—H3AA	109.0	O5—C17—O4	123.1 (2)
C14A—N3A—H3AB	109.0	O5—C17—C18	118.8 (2)
C14A—N3A—C16A	113.13 (16)	O1B—C10B—C2B	115.1 (2)
C8A—C9A—C3A	121.51 (16)	O2B—C10B—O1B	121.0 (2)
C8A—C9A—C4A	118.54 (17)	O2B—C10B—C2B	123.9 (2)
C4A—C9A—C3A	119.95 (17)	O1A—C10A—C2A	115.14 (19)
N1A—C8A—C9A	118.88 (16)	O2A—C10A—O1A	120.9 (2)
N1A—C8A—C7A	120.69 (16)	O2A—C10A—C2A	123.9 (2)
C9A—C8A—C7A	120.41 (16)	N2B—C15B—H15C	110.0
N1B—C8B—C9B	119.12 (16)	N2B—C15B—H15D	110.0
N1B—C8B—C7B	120.38 (17)	N2B—C15B—C16B	108.6 (2)
C7B—C8B—C9B	120.50 (16)	H15C—C15B—H15D	108.4
C8B—C9B—C3B	121.28 (16)	C16B—C15B—H15C	110.0
C4B—C9B—C8B	118.30 (17)	C16B—C15B—H15D	110.0
C4B—C9B—C3B	120.41 (17)	N2B—C13B—H13C	109.9
H3BA—N3B—H3BB	107.8	N2B—C13B—H13D	109.9
C14B—N3B—H3BA	109.0	N2B—C13B—C14B	109.1 (2)
C14B—N3B—H3BB	109.0	H13C—C13B—H13D	108.3
C14B—N3B—C16B	113.09 (18)	C14B—C13B—H13C	109.9
C16B—N3B—H3BA	109.0	C14B—C13B—H13D	109.9
C16B—N3B—H3BB	109.0	N3A—C16A—C15A	110.78 (18)
N2A—C6A—C7A	123.27 (18)	N3A—C16A—H16A	109.5
N2A—C6A—C5A	119.65 (17)	N3A—C16A—H16B	109.5
C7A—C6A—C5A	117.03 (17)	C15A—C16A—H16A	109.5
C8A—C7A—H7A	119.6	C15A—C16A—H16B	109.5
C6A—C7A—C8A	120.88 (17)	H16A—C16A—H16B	108.1
C6A—C7A—H7A	119.6	N3A—C14A—C13A	109.98 (18)
C8B—C7B—H7B	119.3	N3A—C14A—H14A	109.7
C6B—C7B—C8B	121.35 (18)	N3A—C14A—H14B	109.7
C6B—C7B—H7B	119.3	C13A—C14A—H14A	109.7
O3A—C3A—C9A	121.85 (17)	C13A—C14A—H14B	109.7
O3A—C3A—C2A	122.73 (18)	H14A—C14A—H14B	108.2
C2A—C3A—C9A	115.42 (17)	H9A—O9—H9B	104.5
C3A—C2A—C10A	121.34 (19)	C11B—C12B—H12D	109.5
C1A—C2A—C3A	120.20 (17)	C11B—C12B—H12E	109.5
C1A—C2A—C10A	118.45 (18)	C11B—C12B—H12F	109.5
C9A—C4A—H4A	120.2	H12D—C12B—H12E	109.5
C5A—C4A—C9A	119.70 (17)	H12D—C12B—H12F	109.5
C5A—C4A—H4A	120.2	H12E—C12B—H12F	109.5
N2B—C6B—C7B	122.18 (18)	N3B—C14B—C13B	110.58 (18)
N2B—C6B—C5B	121.09 (17)	N3B—C14B—H14C	109.5
C7B—C6B—C5B	116.58 (17)	N3B—C14B—H14D	109.5
C3B—C2B—C10B	121.56 (19)	C13B—C14B—H14C	109.5
C1B—C2B—C3B	120.43 (17)	C13B—C14B—H14D	109.5
C1B—C2B—C10B	118.01 (19)	H14C—C14B—H14D	108.1
O3B—C3B—C9B	121.72 (18)	N3B—C16B—C15B	111.45 (19)
O3B—C3B—C2B	123.02 (18)	N3B—C16B—H16C	109.3
C2B—C3B—C9B	115.26 (17)	N3B—C16B—H16D	109.3
N1B—C1B—C2B	124.01 (17)	C15B—C16B—H16C	109.3
N1B—C1B—H1BA	118.0	C15B—C16B—H16D	109.3
C2B—C1B—H1BA	118.0	H16C—C16B—H16D	108.0
N1A—C1A—C2A	123.90 (17)	C11A—C12A—H12A	109.5
N1A—C1A—H1AA	118.0	C11A—C12A—H12B	109.5
C2A—C1A—H1AA	118.0	C11A—C12A—H12C	109.5
F1A—C5A—C6A	117.41 (17)	H12A—C12A—H12B	109.5
C4A—C5A—F1A	119.11 (18)	H12A—C12A—H12C	109.5
C4A—C5A—C6A	123.42 (17)	H12B—C12A—H12C	109.5
C9B—C4B—H4B	120.1	O8—C19—C20	120.7 (3)
C5B—C4B—C9B	119.78 (18)	O7—C19—O8	121.7 (3)
C5B—C4B—H4B	120.1	O7—C19—C20	117.6 (3)
F1B—C5B—C6B	118.19 (17)	C17—C18—H18A	109.5
F1B—C5B—C4B	118.25 (18)	C17—C18—H18B	109.5
C4B—C5B—C6B	123.48 (17)	C17—C18—H18C	109.5
N2A—C13A—H13A	109.8	H18A—C18—H18B	109.5
N2A—C13A—H13B	109.8	H18A—C18—H18C	109.5
N2A—C13A—C14A	109.17 (18)	H18B—C18—H18C	109.5
H13A—C13A—H13B	108.3	C19—C20—H20A	109.5
C14A—C13A—H13A	109.8	C19—C20—H20B	109.5
C14A—C13A—H13B	109.8	C19—C20—H20C	109.5
N1A—C11A—H11A	109.3	H20A—C20—H20B	109.5
N1A—C11A—H11B	109.3	H20A—C20—H20C	109.5
N1A—C11A—C12A	111.70 (18)	H20B—C20—H20C	109.5
H11A—C11A—H11B	107.9		
			
O3A—C3A—C2A—C1A	179.57 (18)	C4A—C9A—C8A—C7A	−0.6 (3)
O3A—C3A—C2A—C10A	0.6 (3)	C4A—C9A—C3A—O3A	−0.9 (3)
N1A—C8A—C7A—C6A	177.68 (16)	C4A—C9A—C3A—C2A	178.98 (16)
N1B—C8B—C9B—C3B	0.2 (3)	C6B—N2B—C15B—C16B	−145.3 (2)
N1B—C8B—C9B—C4B	179.12 (16)	C6B—N2B—C13B—C14B	145.3 (2)
N1B—C8B—C7B—C6B	−179.32 (16)	C3B—C9B—C4B—C5B	179.30 (18)
N2A—C6A—C7A—C8A	179.12 (17)	C3B—C2B—C1B—N1B	0.5 (3)
N2A—C6A—C5A—F1A	−1.6 (3)	C3B—C2B—C10B—O1B	0.0 (3)
N2A—C6A—C5A—C4A	−178.95 (19)	C3B—C2B—C10B—O2B	179.9 (2)
N2A—C13A—C14A—N3A	55.1 (2)	C1B—N1B—C8B—C9B	1.8 (3)
N2A—C15A—C16A—N3A	−56.6 (2)	C1B—N1B—C8B—C7B	−177.76 (17)
N2B—C6B—C5B—F1B	0.3 (3)	C1B—N1B—C11B—C12B	94.2 (2)
N2B—C6B—C5B—C4B	−176.5 (2)	C1B—C2B—C3B—O3B	−178.19 (18)
N2B—C15B—C16B—N3B	−55.9 (3)	C1B—C2B—C3B—C9B	1.5 (3)
N2B—C13B—C14B—N3B	55.6 (3)	C1B—C2B—C10B—O1B	−179.39 (19)
C9A—C8A—C7A—C6A	−0.7 (3)	C1B—C2B—C10B—O2B	0.5 (3)
C9A—C3A—C2A—C1A	−0.3 (3)	C1A—N1A—C8A—C9A	0.4 (3)
C9A—C3A—C2A—C10A	−179.30 (16)	C1A—N1A—C8A—C7A	−178.04 (16)
C9A—C4A—C5A—F1A	−177.18 (16)	C1A—N1A—C11A—C12A	94.8 (2)
C9A—C4A—C5A—C6A	0.1 (3)	C1A—C2A—C10A—O1A	−177.65 (18)
C8A—N1A—C1A—C2A	−1.6 (3)	C1A—C2A—C10A—O2A	1.4 (3)
C8A—N1A—C11A—C12A	−79.1 (2)	C5A—C6A—C7A—C8A	1.6 (3)
C8A—C9A—C3A—O3A	179.32 (17)	C4B—C9B—C3B—O3B	−1.0 (3)
C8A—C9A—C3A—C2A	−0.8 (2)	C4B—C9B—C3B—C2B	179.32 (16)
C8A—C9A—C4A—C5A	0.9 (3)	C13A—N2A—C6A—C7A	127.7 (2)
C8B—N1B—C1B—C2B	−2.3 (3)	C13A—N2A—C6A—C5A	−54.8 (3)
C8B—N1B—C11B—C12B	−80.9 (2)	C13A—N2A—C15A—C16A	63.6 (2)
C8B—C9B—C3B—O3B	177.84 (17)	C11A—N1A—C8A—C9A	174.25 (16)
C8B—C9B—C3B—C2B	−1.8 (3)	C11A—N1A—C8A—C7A	−4.1 (3)
C8B—C9B—C4B—C5B	0.4 (3)	C11A—N1A—C1A—C2A	−175.62 (18)
C8B—C7B—C6B—N2B	175.47 (18)	C11B—N1B—C8B—C9B	176.91 (17)
C8B—C7B—C6B—C5B	0.0 (3)	C11B—N1B—C8B—C7B	−2.7 (3)
C9B—C8B—C7B—C6B	1.1 (3)	C11B—N1B—C1B—C2B	−177.51 (18)
C9B—C4B—C5B—F1B	−175.99 (17)	C15A—N2A—C6A—C7A	−26.0 (3)
C9B—C4B—C5B—C6B	0.7 (3)	C15A—N2A—C6A—C5A	151.4 (2)
C6A—N2A—C13A—C14A	140.2 (2)	C15A—N2A—C13A—C14A	−63.6 (2)
C6A—N2A—C15A—C16A	−140.6 (2)	C10B—C2B—C3B—O3B	2.4 (3)
C7A—C6A—C5A—F1A	175.97 (17)	C10B—C2B—C3B—C9B	−177.92 (17)
C7A—C6A—C5A—C4A	−1.4 (3)	C10B—C2B—C1B—N1B	179.95 (18)
C7B—C8B—C9B—C3B	179.81 (17)	C10A—C2A—C1A—N1A	−179.45 (18)
C7B—C8B—C9B—C4B	−1.3 (3)	C15B—N2B—C6B—C7B	−15.7 (3)
C7B—C6B—C5B—F1B	175.79 (18)	C15B—N2B—C6B—C5B	159.6 (2)
C7B—C6B—C5B—C4B	−0.9 (3)	C15B—N2B—C13B—C14B	−63.6 (2)
C3A—C9A—C8A—N1A	0.8 (2)	C13B—N2B—C6B—C7B	132.7 (2)
C3A—C9A—C8A—C7A	179.21 (16)	C13B—N2B—C6B—C5B	−52.0 (3)
C3A—C9A—C4A—C5A	−178.93 (17)	C13B—N2B—C15B—C16B	63.2 (2)
C3A—C2A—C1A—N1A	1.5 (3)	C16A—N3A—C14A—C13A	−51.4 (2)
C3A—C2A—C10A—O1A	1.4 (3)	C14A—N3A—C16A—C15A	52.7 (3)
C3A—C2A—C10A—O2A	−179.5 (2)	C14B—N3B—C16B—C15B	50.9 (3)
C4A—C9A—C8A—N1A	−179.00 (16)	C16B—N3B—C14B—C13B	−50.5 (3)

### 4-(3-Carboxy-1-ethyl-6-fluoro-4-oxo-1,4-dihydroquinolin-7-yl)piperazin-1-ium acetate sesquihydrate Hydrogen-bond geometry (Å, º)

**Table:**

*D*—H···*A*	*D*—H	H···*A*	*D*···*A*	*D*—H···*A*
O1*A*—H1*A*···O3*A*	0.82	1.75	2.520 (2)	155
O1*B*—H1*B*···O3*B*	0.82	1.77	2.533 (2)	154
N3*A*—H3*AA*···O6	0.89	1.84	2.709 (3)	164
N3*A*—H3*AB*···O4	0.89	1.80	2.673 (3)	165
N3*B*—H3*BA*···O10	0.89	2.45	3.161 (4)	137
N3*B*—H3*BB*···O8	0.89	1.80	2.679 (3)	171
C13*B*—H13*C*···F1*B*	0.97	2.27	2.881 (3)	120
C13*A*—H13*A*···F1*A*	0.97	2.34	2.867 (3)	114
O6—H6*A*···O7	0.85	1.82	2.664 (5)	172
O6—H6*B*···O9	0.85	1.92	2.706 (5)	153
O9—H9*A*···O8^i^	0.85	1.91	2.706 (4)	156
O9—H9*B*···O5^i^	0.85	1.93	2.733 (3)	158
O10—H10*A*···O4	0.85	2.00	2.734 (3)	145
O10—H10*B*···O5^i^	0.85	1.99	2.811 (3)	161

Symmetry code: (i) *x*+1, *y*, *z*.

## References

[bb1] Abidi, M. Z., Ledeboer, N., Banerjee, A. & Hari, P. (2016). *Diagn. Microbiol. Infect. Dis.* **85**, 116–120. 10.1016/j.diagmicrobio.2016.01.005 26906191

[bb2] Barry, A. L., Jones, R. N., Thornsberry, C., Ayers, L. W., Gerlach, E. H. & Sommers, H. M. (1984). *Antimicrob. Agents Chemother.* **25**, 633–637. 10.1128/aac.25.5.633 PMC185603 6233935

[bb3] Dolomanov, O. V., Bourhis, L. J., Gildea, R. J., Howard, J. A. K. & Puschmann, H. (2009). *J. Appl. Cryst.* **42**, 339–341.

[bb4] Ferrazzi, E., Peracchi, M., Biasolo, M. A., Faggionato, O., Stefanelli, S. & Palu’, G. (1988). *Biochem. Pharmacol.* **37**, 1885–1886. 10.1016/0006-2952(88)90495-9 2837249

[bb5] Goldstein, E. J. (1987). *Am. J. Med.* **82**, 3–17. 10.1016/0002-9343(87)90612-7 3111257

[bb6] Grangé, J. D., Roulot, D., Pelletier, G., Pariente, É. A., Denis, J., Ink, O., Blanc, P., Richardet, J. P., Vinel, J. P., Delisle, F., Fischer, D., Flahault, A. & Amiot, X. (1998). *J. Hepatol.* **29**, 430–436. 10.1016/s0168-8278(98)80061-5 9764990

[bb7] Groom, C. R., Bruno, I. J., Lightfoot, M. P. & Ward, S. C. (2016). *Acta Cryst.* B**72**, 171–179. 10.1107/S2052520616003954 PMC4822653 27048719

[bb8] Gunnam, A. & Nangia, A. K. (2023). *Cryst. Growth Des.* **23**, 4198–4213.

[bb9] Jiang, X., Yuan, S., Yang, J., Liao, H., Wu, H., Jiang, B. & Qian, K. (2025). *J. Mol. Struct.* **1353**, 144732.

[bb10] Jiao, L. T., Yang, D. Z., Zhang, L., Yang, S. Y., Du, G. H. & Lu, Y. (2021). *J. Mol. Struct.* **1223**, 128865.

[bb11] O’Malley, C., McArdle, P. & Erxleben, A. (2022). *Cryst. Growth Des.* **22**, 3060–3071. 10.1021/acs.cgd.1c01509 PMC9073931 35529070

[bb12] Pandeya, S. N., Sriram, D., Nath, G. & De Clercq, E. (2000). *Eur. J. Med. Chem.* **35**, 249–255. 10.1016/s0223-5234(00)00125-2 10758286

[bb13] Rasulov, A., Torambetov, B., Alimnazarov, B., Kadirova, S., Suyunov, J., Nazarov, Y. & Ashurov, J. (2024). *IUCrData* **9**, x240813. 10.1107/S2414314624008137 PMC11375602 39247080

[bb14] Rasulov, A., Torambetov, B., Suyunov, J., Eshkaraev, S., Kholmurodova, L., Babamuratov, B. & Ashurov, J. (2025). *Acta Cryst.* E**81**, 944–947. 10.1107/S205698902500787X PMC12498055 41059319

[bb15] Rigaku OD (2021). *CrysAlis PRO*. Rigaku Oxford Diffraction, Yarnton, England.

[bb16] Sheldrick, G. M. (2015*a*). *Acta Cryst.* A**71**, 3–8.

[bb17] Sheldrick, G. M. (2015*b*). *Acta Cryst.* C**71**, 3–8.

[bb20] Spackman, P. R., Turner, M. J., McKinnon, J. J., Wolff, S. K., Grimwood, D. J., Jayatilaka, D. & Spackman, M. A. (2021). *J. Appl. Cryst.* **54**, 1006–1011. 10.1107/S1600576721002910 PMC8202033 34188619

[bb21] Wang, Y., Sun, L., Wang, W. & Yan, L. (2005). *Chin. J. Struct. Chem.* **24**, 1359–1362.

[bb22] Zeng, X., Zheng, C., Qiu, S., Jiang, X., Wang, X., Qian, K., Pan, R., Yang, J. & Ma, Y. (2024). *Cryst. Growth Des.* **24**, 4416–4427.

[bb23] Zhang, X., Zhang, Y., Liu, L., Ye, Z., Zhang, X., Liu, Y., Wang, T., Geng, H., Xia, Y. & Gao, N. (2025). *J. Mol. Struct.* **1348**, 143485.

